# Social representations of pregnant women about high-risk pregnancy: repercussions for prenatal care*

**DOI:** 10.1590/1980-220X-REEUSP-2022-0463en

**Published:** 2023-10-13

**Authors:** Alexandre Aguiar Pereira, Ivaneide Leal Ataíde Rodrigues, Laura Maria Vidal Nogueira, Iací Proença Palmeira, Heliana Helena de Moura Nunes, Erlon Gabriel Rego de Andrade, Fabiane Oliveira da Silva

**Affiliations:** 1 Universidade do Estado do Pará, Escola de Enfermagem Magalhães Barata, Programa de Pós-Graduação em Enfermagem, Belém, PA, Brazil.; 2 Universidade do Estado do Pará, Escola de Enfermagem Magalhães Barata, Departamento de Enfermagem Comunitária, Belém, PA, Brazil.; 3 Fundação Santa Casa de Misericórdia do Pará, Programa de Pós-Graduação em Gestão e Saúde na Amazônia, Belém, PA, Brazil.

**Keywords:** Pregnancy, High-Risk, Prenatal Care, Psychology, Social, Maternal-Child Nursing, Secondary Care, Embarazo de Alto Riesgo, Atención Prenatal, Psicología Social, Enfermería Maternoinfantil, Atención Secundaria de Salud, Gravidez de Alto Risco, Cuidado Pré-Natal, Psicologia Social, Enfermagem Materno-Infantil, Atenção Secundária à Saúde

## Abstract

**Objective:**

To analyze the Social Representations of pregnant women about high-risk pregnancy and its repercussions for prenatal care.

**Method:**

Qualitative study, based on the procedural aspect of the Theory of Social Representations, carried out with 62 high-risk prenatal pregnant women at a High Complexity Maternity, in Belém, PA, Brazil. Data from semi-structured interviews were processed by the software *Interface de R pour les Analyzes Multidimensionnelles de Textes et de Questionnaires*.

**Results:**

Four categories emerged, in which three dimensions of the Theory composing the genesis of Social Representations are considered: the affective dimension, the biological dimension and the sociocultural dimension.

**Conclusion:**

Affects, negative feelings, and adaptations were revealed, with high-risk pregnancy being represented as an unusual and uncomfortable event, influenced by common sense and science, communication means, and dialogues with health professionals, with family support being considered of paramount importance. and prenatal care a propitious moment for establishing bonds with the health professional, seen as essential for their adherence to the care offered.

## RESUMO

**Objetivo:**

Analisar as Representações Sociais de mulheres grávidas sobre a gestação de alto risco e suas repercussões para assistência pré-natal.

**Método:**

Estudo qualitativo, pautado na vertente processual da Teoria das Representações Sociais, realizado com 62 gestantes do pré-natal de alto risco de uma Maternidade de Alta Complexidade, em Belém, PA, Brasil. Os dados das entrevistas semiestruturadas foram processados pelo *software Interface de R pour les Analyses Multidimensionnelles de Textes et de Questionnaires*.

**Resultados:**

Emergiram quatro categorias, nas quais consideram-se três dimensões da Teoria que compuseram a gênese das Representações Sociais, quais sejam: a dimensão afetiva, a dimensão biológica e a dimensão sociocultural.

**Conclusão:**

Desvelaram-se afetos, sentimentos negativos e adaptações, representando-se a gestação de alto risco como evento inusitado e desconfortável, influenciado pelo senso comum e ciência, meios de comunicação e diálogos com os profissionais de saúde, sendo o suporte familiar considerado de suma importância e o pré-natal um momento propício para estabelecimento de vínculos com o profissional de saúde, visto como fundamental para sua adesão aos cuidados ofertados.

## RESUMEN

**Objetivo:**

Analizar las Representaciones Sociales de gestantes sobre el embarazo de alto riesgo y sus repercusiones para el prenatal.

**Método:**

Estudio cualitativo, basado en el aspecto procedimental de la Teoría de las Representaciones Sociales, realizado con 62 gestantes en la atención prenatal de alto riesgo en una Maternidad de Alta Complejidad, en Belém, PA, Brasil. Los datos de las entrevistas semiestructuradas fueron procesados por el *software Interface de R pour les Analyses Multidimensionnelles de Textes et de Questionnaires*.

**Resultados:**

Surgieron cuatro categorías, en las que se consideraron tres dimensiones de la Teoría que compusieran la génesis de las Representaciones Sociales, a saber: la dimensión afectiva, la dimensión biológica y la dimensión sociocultural.

**Conclusión:**

Se revelaron afectos, sentimientos negativos y adaptaciones, representando el embarazo de alto riesgo como un evento insólito e incómodo, influenciado por el sentido común y la ciencia, los medios de comunicación y diálogos con profesionales de la salud, considerándose de suma importancia el apoyo familiar y el control prenatal, un momento propicio para establecer vínculos con el profesional de la salud, visto como fundamental para su adhesión a los cuidados ofrecidos.

## INTRODUCTION

Pregnancy is a physiological phenomenon, a moment of great physical and emotional changes for women. Its evolution is generally uneventful. However, about 20% of pregnancies are likely to have an unfavorable outcome for the mother-baby binomial^(1)^.

In this context, high-risk pregnancy (HRP) is configured, which is understood as a series of unexpected clinical, obstetric, and social conditions, associated with real or potential danger to the pregnancy, requiring changes in lifestyle, technical support, and/or hospitalization^(2)^, specialized follow-up, early identification of problems related to clinical, socioeconomic and demographic conditions, and provision of satisfactory perinatal diagnostic and therapeutic procedures^(1)^.

With the completion of the Millennium Development Goals in 2015, it was found that the 70% reduction, since 1990, of the Maternal Mortality Ratio (MMR) was not achieved in most countries, including Brazil, which reduced it by around 50%. This year, the five Brazilian regions showed a decreasing trend in the MMR, except for the North Region, with Pará having one of the highest, 72.9/100,000 live births^(3)^, reinforcing the extent to which maternal mortality represents a challenge for the State.

In 2021, the country recorded a rate of 107.53 deaths per 100,000 live births, according to preliminary data from the Ministry of Health mapped by the Brazilian Obstetric Observatory. In 2019, this rate was 55.31 per 100,000 live births, while in 2020, this same number jumped to 71.97 deaths, which already represented an increase of almost 25% compared to the previous year^(4)^.

Due to the high rates of maternal and child mortality, prenatal care has been highlighted in Brazil, with the establishment of a care plan, sharing of actions, teamwork, and effective negotiation of care for the pregnant woman^(5)^, with care of pregnant women at risk in high-risk prenatal care (*PNAR*) by a multidisciplinary team, which includes the nurse^(6)^, being recommended. However, although public policies aimed at high-risk pregnancies advance in the provision of care services, these latter are based on the biomedical model^(7)^.

Due to its complexity, the HRP should not be understood only regarding biological aspects, such as the determination of causes, consequences and treatment of intercurrences, but also its social dynamics, which involves the woman and her construction processes of knowledge, behaviors, beliefs, communication, and the meanings that add value to the phenomena they will face during this pregnancy^(8)^.

In a woman’s life cycle, pregnancy is a period that demands changes in the social role, personal readjustments, physical and emotional adaptations, profoundly affecting them, their partners and relatives^(8)^, especially when it is associated with complications, configuring itself as a phenomenon of Social Representations (SR).

The Social Representations Theory (SRT) shows SRs as common sense knowledge, practical knowledge built and shared in society or group, with the purpose of building a common reality for all individuals belonging to it, mediating social relations with the macro (society) and micro (individual) spheres, such as behavior, communications, and identity^(9)^.

The object to generate SR shall have cultural and social relevance, which means being in the individuals’ daily lives, as a practical experience of the group^(10)^. Therefore, it is understood that investigating HRP, based on the representations constructed and shared socially by pregnant women, can support the understanding of the repercussions of these SRs for adherence to the *PNAR*. Thus, the goal established was to analyze the Social Representations of pregnant women about high-risk pregnancy and its repercussions for prenatal care.

## METHOD

### Design of Study

Qualitative study, based on the procedural aspect of the SRT, a theory of a psychosocial nature that allowed revealing the reality of the other, accessing their ways of acting and thinking about the phenomenon, understanding the meanings attributed and explanations produced, allowing the apprehension of behaviors adopted in daily life^(10)^. The construction of the manuscript followed the recommendations of the *Consolidated Criteria for Reporting Qualitative Research* ^(11)^.

### Local

High Risk Prenatal Service (*PNAR*), from the Santa Casa de Misericórdia do Pará Foundation (*FSCMPA*), located in the city of Belém, PA, Brazil, a reference in the State, which serves pregnant women referred from the 144 municipalities of Pará, from the primary and specialized network, providing care by a multidisciplinary team.

### Population and Selection Criteria

Participants were 62 pregnant women, selected for convenience, assisted in the morning and afternoon, adopting sampling through theoretical saturation. Inclusion criteria were: pregnant women over 18 years of age, registered in the *PNAR*, regardless of gestational age (GA) and number of pregnancies, who had physical and mental conditions to respond to the interview.

The following were excluded: pregnant women undergoing PNAR screening, those with a diagnosis of fetal life incompatibility, due to the emotional implications that involve these pregnancies, and pregnant women under 18 years of age, since they are assisted in the care and protection program for children and adolescents (PROPAZ), commonly victims of sexual violence, which has a strong emotional impact.

### Data Collection

Data production took place from May to July 2021, carried out exclusively by the researcher in charge, who did not have an institutional, labor, or personal relationship with the institution and/or professionals who worked there. The researcher was introduced to the *PNAR* team by the management of the Outpatient Clinic, to facilitate the approach of the pregnant women to the place and the assignment of space to carry out the individual interviews, aiming at keeping participants’ comfort and privacy.

On consecutive days, the research was presented to pregnant women gathered in small groups in the *PNAR* environments and, with those who met the eligibility criteria, carefully verified by the researcher in charge, an individual invitation was made to participate, approaching them before or after consultations so as not to interfere with their care. Upon acceptance, the interviews were carried out on the same day or were scheduled to take place according to the availability of the participants and the researcher, in a previously reserved room.

The interviews lasted an average of 30 minutes and were recorded in MP3 format, with prior authorization. A semi-structured form, prepared by the researcher, was used to characterize the sociodemographic and obstetric profile of pregnant women, seeking to know their belonging, and to explore the object of study, through open questions.

### Data Analysis and Treatment

The transcribed testimonies constituted the *corpus*, posteriorly processed through the *software* IRaMuTeQ (*Interface de R pour les Analyzes Multidimensionnelles de Textes et de Questionnaires*), version 0.7, alpha 2, textual data processing and analysis software. Descending Hierarchical Classification (DHC) was chosen, considering the words that presented chi-square (χ^2^) with significance values of p ≤ 0.01^(12)^. The software organized the dendrogram, relating the classes, and provided results from the description of each of them, mainly by the vocabulary present in the text segments (TS), allowing later organization of the classes in thematic categories.

The analysis and interpretation were based on the concepts of Anchoring, which seeks to add an idea, hitherto unknown, in a familiar context, and Objectification, which seeks to turn the immaterial into material, that is, the “materialization” of the abstract, fundamental processes for the elaboration of SR^(9)^.

### Ethical Aspects

Resolution No. 466/2012 was complied with, approved by the Research Ethics Committee of the Nursing School Magalhães Barata, Universidade do Estado do Pará (*UEPA*), under opinion No. 4.397.267/2020, and of the *FSCMPA*, opinion No. 4.428.991/2020. All participants signed the Informed Consent Form prior to the interviews, declaring their formal acceptance, at which point the researcher clarified doubts regarding the form of participation, the risks and benefits of the research. Identity secrecy was guaranteed through the use of an alphanumeric code formed by the letter G, for “pregnant woman”, followed by the cardinal number that indicates the order of the interviews.

## RESULTS

### Sociodemographic and Obstetric Characterization of the Participants

Starting from the understanding that for studies that discuss the SR it is important to know the participants’ belonging, the pregnant women who participated in the study were characterized, for the understanding of who they were and where they spoke from, apprehending their nuances and peculiarities, as well as the context that outlined what they said about the object of study^(10)^.

Among the 62 participants, 37 (59.7%) lived in the Metropolitan Region, mainly in the capital Belém (24/38.7%), and 25 (40.3%) in other municipalities in Pará. The age group from 18 to 28 years old (30/48.4%) predominated, 37 were (59.7%) in a common-law marriage, 38 (61.2%) were evangelicals, with finished high school (30/48.4%), and 48 (77.4%) self-described them as brown. The majority (36/58.1%) were housewives, with a monthly personal/family income of one minimum wage (26/41.9%).

As for the obstetric characteristics, in the GA, 20 to 30 weeks predominated (28/45.2%), the majority being multigravida and multiparous (46/74.2%). Regarding the diagnoses associated with gestational risk, maternal pathologies predominated (29/46.8%), especially hypertensive diseases (12/19.3%) and gestational diabetes (9/14.5%), followed, respectively, by fetal pathologies and malformations (18/29%), such as omphalocele, hydrocephalus, congenital heart disease, among others, and bad obstetric history (15/24.2%), such as abortions, premature births, intrauterine fetal death, and repeat cesarean sections.

### Presentation of Classes/Categories

**Figure 1:**
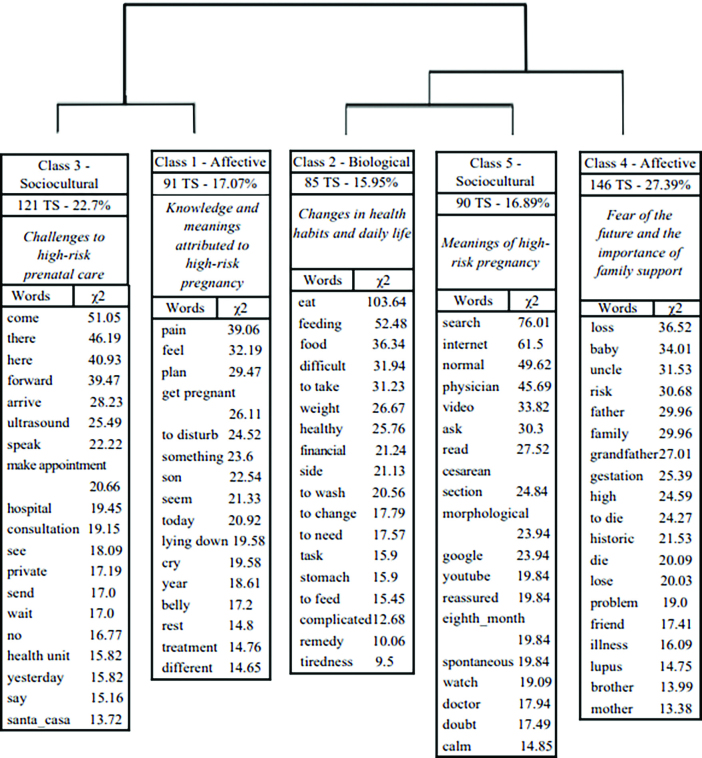
Dendrogram of Descending Hierarchical Classification of *corpus* “Social representations of pregnant women about high-risk pregnancy”. Belém, Pará, Brazil, 2022.

Sixty-two texts, which after being run in the IRaMuTeQ unfolded into 705 TS, were organized from the *corpus*. A total of 24,108 occurrences of words were analyzed, an average of 34.19 occurrences per segment. From the DHC, the software generated five classes using a dendrogram, with 75.60% of the statements being used (Figure 1).

For a better presentation of the results and discussion in light of the SRT, classes were identified according to three dimensions, through the contexts that emerged from the words with greater significance (chi-square), described according to the SRT conceptions about the genesis of the different constituent dimensions of the subject^(9,10)^: affective (performance of feelings, affections and values); biological (cerebral and physiological functioning) and sociocultural (influence of the social context, language, beliefs, and communication), composing the SR on the HRP.

Thus, classes 1 and 4 composed the affective dimension, class 2 the biological dimension, and classes 3 and 5 the sociocultural dimension, allowing the formation of four categories: Knowledge and meanings attributed to high risk pregnancy (affective dimension); Fear of the future and importance of family support (affective dimension); Changes in health and daily habits (biological dimension); and high-risk prenatal care: challenges and meanings of high-risk pregnancy (sociocultural dimension).

### Category 1: Knowledge and Meanings Attributed to High-Risk Pregnancy (Affective Dimension)

This category emerged from class 1, formed by 91 TS (17.07%) and 16 representative words (with greater frequency – f) and with greater strength (chi-square test – χ^2^). It presents the understanding and imaginary attributed to HRP, based on their understanding of it and experiences acquired during pregnancy, as well as the biopsychosocial repercussions on their daily lives, mentioned as inherent to the gestational risk, also highlighting the reality in which they lived, the processes experienced by them and knowledge about HRP related to physical/emotional manifestations and how they interfered in their routine.

The strength of words pain (χ^2^ = 39.06), feel (χ^2^ = 32.19), plan (χ^2^ = 29.47), get pregnant (χ^2^ = 26.11) and to disturb (χ^2^ = 24.52) illustrated the meanings of this pregnancy, portrayed as closely linked to what they felt and experienced. They reported the physical pain as negative for perinatal well-being and health, causing discomfort for the pregnant woman and making it less pleasant, explaining the complications generated and their multiple effects:

*What has changed is because, in this one* [referring to the current pregnancy]*, I stand still! I feel pain everywhere, in my back, in the other I used to ride a bicycle after get pregnant; in this one, I can’t even walk on foot, I stand up for a little while and feel dizzy. (G16)*

*My life, my routine, has changed because I get stressed, I don’t have much patience, I cry, I start to feel pain everywhere, throughout my body. My life is being sat or lying down, even to come here I have to plan before. They ordered and I have to come, but it’s tiring, it bothers me, I get sore. (G40)*

The word cry (χ^2^ = 19.58) showed how significant the high-risk diagnosis can be for them, generating negative feelings and sadness. When experiencing this process, they dealt with the characteristic transformations of pregnancy that were enhanced in view of the gestational risk:

*The girls* [from the nursing team] *talked to me a lot on the first day! I just kept crying, I looked at my children and the cry came, and that was harming the child, because I felt a lot of pain in the uterus. (G60)*

*[...] My pregnancy here is very different from the others, everything is different! I already felt very sick in this one, I feel malaise, I vomit, I cry, it’s terrible! (G2)*

Faced with the meanings of the HRP, they built their representations based on negative affects and feelings, with pain denoting limitation and discomfort, and cry denoting sadness, giving meaning to the object. Based on this view, HRP was represented as a set of feelings and sensations, endowed with singularities and subjectivities distinct from those previously experienced.

### Category 2: Fear of the Future and Importance of Family Support (Affective Dimension)

Class 4, formed by 146 TS (27.39%) and 19 representative words, originated the category about the fears manifested by them in relation to the possibility of dying, the loss of the baby and relive negative experiences, in addition to the role of the family as a support for times of difficulty imposed by the unusual situation.

The testimonies showed HRP associated with the unfavorable progression of pregnancy, directly affecting the health of the pregnant woman and the fetus. They began to think about an uncertain future, the possibility of death, making them feel anxious and powerless to save the baby:

*I have to be careful, because of high blood pressure, I may die! So far, my greatest fear, I don’t know, is of dying, of something happening to me, if my pregnancy does not go to term. (G50)*

*It’s this thing that can’t get out of my mind, saying that I won’t have the strength to have this baby, that I’ll die in the operating room, because I intend to have an operation, I’m suffering from this, [...] I told the doctor that my heart is accelerated, I think this anxiety is killing me. (G11)*

It should be noted that the explanations were anchored in traumatic memories experienced in other pregnancies, associated with the death/loss of the baby and the impossibility of sustaining the pregnancy, representing the fear of loss and the possibility of reliving negative experiences, expressed in the words loss (χ^2^ = 36 .52), baby (χ^2^ = 34.01) and historic (χ^2^ = 21.53):

*Each one is a different experience, which was just a baby! I’m afraid because it’s new, when it’s time to give birth, even today I’m afraid of having them* [referring to babies]*, we do not know how it will be. (G12)*

*Because I was very worried about it happening again, but it’s been very different. Of my first* [baby]*, I lost, my belly didn’t grow. Now I need a more specific treatment than in the other* [pregnancies]*, because I already have a historic of loss. (G7)*

When envisioning the future of pregnancy and childbirth, they highlighted the fear of the baby being born with health problems, expressed in the words baby (χ^2^ = 34.01), problem (χ^2^ = 19.0) and illness (χ^2^ = 16.09). They elaborated an imaginary of a healthy and perfect baby during pregnancy, represented by fantasies and desires, an image abruptly broken with the high-risk diagnosis, making them reframe the pregnancy as a whole and the baby’s imagery construction:

*[...] I was worried about the baby! I was worried, like, it’s not a pregnancy I planned, I didn’t want it to be that way, but I didn’t refuse either, even when I found out about the risks, I’m in love with the child, so I don’t want to do him any harm and I don’t want him to have any problem. (G24)*

*I was very scared! Because I expected everything to be normal, now I don’t know how it will be, having a child with an illness, because I already have a son and he is very young too. (G59)*

Faced with concern and fears, they highlighted the importance of the family, seen as a foundation for coping with difficulties. Words like uncle (χ^2^ = 31.53), father (χ^2^ = 29.96), family (χ^2^ = 29.96), grandfather (χ^2^ = 27.01), brother (χ^2^ = 13.99) and mother (χ^2^ = 13.38), presented their family support network, reiterating that it brought a sense of safety and tranquility. The effects of this pregnancy on them and their social web were evident, especially those closest to them, showing the family as a support for coping with the adaptations, difficulties and re-signification of this reality:

*My mother is everything to me, she is cooking my food, she is a cook, so she is making healthier food, salads, fruits and changed my diet. My family knows, my husband is out there, everyone supports me. (G48)*

*At first, I didn’t want to talk to my father, grandfather, uncle, cousin, brother, anyone, but then everyone was already supporting me, they called me, texted me, asking how I am, how the baby is, this is so good! At first I was scared, now I’m calm. (G61)*

### Category 3: Changes in Health and Daily Habits (Biological Dimension)

This category emerged from class 2, formed by 85 TS (15.95%) and 18 representative words. It presents changes in health and daily habits based on changes in food, body, household activities, and the need for medication. The words eat (χ^2^ = 103.64), feeding (χ^2^ = 52.48), difficult (χ^2^ = 34.01), weight (χ^2^ = 26.67) and healthy (χ^2^ = 25.76) represented the need to readjust eating habits and weight control to maintain strict care:

*[...] We get stressed, especially since I can’t eat everything, I have to be on a diet, I have to be taking insulin, I get stressed, I get bruises on my arms, it’s complicated, it’s difficult! (G47)*

*[...] So, I had to change everything, cut fat, no junk food, I started eating more fruit, vegetables and having a healthier diet, I lost a lot of weight, now that I’m gaining because of the baby’s growth. (G17)*

The predominance of the reified discourse was identified to build the representations of the object, since the knowledge presented was related to the field of biological and health sciences, with the evocation of prescriptive norms such as taking medicine, healthy eating, among others.

They also highlighted changes in the routine for carrying out household chores, expressed in the words difficult (χ^2^ = 34.01), to wash (χ^2^ = 20.56), to change (χ^2^ = 17.79), task (χ^2^ = 15.9) and tiredness (χ^2^ = 9.5). Anchored in the female role, the possibility of performing household chores, even with pregnancy, was associated with maintaining the female attribute of home care. The impossibility of performing them, in the face of HRP, was associated with something that deviated from normality and brought loss of identity:

*I had to completely change! The first thing the doctor prescribes is rest, which is sometimes moderate or absolute, depending on the detachment* [of the placenta], *then that changes completely, because I can’t help my children, I can’t help much with the housework, I need to rely on other people, in this case, I rely on my mother, it gets much more complicated! (G26)*

*When I do something, I’m already tired! If I sweep a house, to wash dishes, wash clothes, it makes me tiredness, I can’t do almost anything at home. My husband fights with me, tells me to lie down, but I don’t stop, because, in my head, it’s hard to understand, I should do it, not him! (G41)*

### Category 4: High-Risk Prenatal Care: Challenges and Meanings of High-Risk Pregnancy (Sociocultural Dimension)

Class 3, formed by 121 TS (22.7%) and 19 representative words, and class 5, formed by 90 TS (16.89%) and 18 representative words, originated category 4, about perceptions in relation to the *PNAR*, search for information, solving doubts in consultations, and importance of the health professional.

It addressed the challenges imposed for monitoring pregnancy, demonstrated by the words come (χ^2^ = 51.05), there (χ^2^ = 46.19), here (22.7%), arrive (χ^2^ = 28.23), make appointment (χ^2^ = 20.66) and consultation (χ^2^ = 19.15), which represented the paths taken to follow up the *PNAR*. They frequently overcame difficulties to carry out consultations, traveling from other municipalities, usually far away, to arrive on time for the scheduled appointment:

*It is difficult to come here, transportation, financial difficulties! Because the cost to go to Belém is high, there is the question of time, because you have to arrive early to undergo tests and you can never leave early, not everyone can get transport. (G16)*

*We come from there at midnight, we come by bus, we can’t sleep, the road is not good, afraid of something happening on the road. You get here, the consultation is quick, but you have to wait until two or three in the afternoon, then you have to make appointment a return visit, when you get home it’s almost 10 pm. That’s hard for me, but I have to come! (G4)*

In this context, it was evident that the social aspect stood out over the biological and emotional issues involving the HRP follow-up, because despite the difficulties and transformations, they understood the importance of consultations for the binomial. The contents of classes 5 and 3 complemented each other, describing the difficulties and understanding the need for specialized follow-up, with the *PNAR* being seen as a generator of information and concerns about themes related to pregnancy.

The words search (χ^2^ = 76.01), internet (χ^2^ = 61.5), video (χ^2^ = 33.82), read (χ^2^ = 27.52), *google* (χ^2^ = 23.94), *youtube* (χ^2^ = 19.84) and watch (χ^2^ = 19.09) showed the other means they sought to obtain information about the pregnancy. The extent of the presence of digital sources in their daily lives was observed, allowing quick access to information, as well as influencing the formation of knowledge, affections, and attitudes. However, they also understood that they have to filter what they access, since the digital medium could bring wrong information:

*Sometimes, I follow through the videos, the internet, YouTube! I’m not afraid, I like to read and watch to learn more about my pregnancy. (G32)*

*I like to search a lot on the internet, even Google! Sometimes, I’m in doubt about certain things, I go online, start reading, watch videos, then, when I come to the consultations, I already know if everything is fine, if it’s normal, if it’s changed, that’s why I really like to be informed! (G55)*

In this change of conception, the interest in information about pregnancy moved away from the consensual universe, present in the mass media, and migrated to reified knowledge, of prescriptive care, guided by professionals, with care practices being influenced by both common sense and scientific knowledge.

Thus, the words ask (χ^2^ = 30.3), reassured (χ^2^ = 19.84) and doubt (χ^2^ = 17.49) indicated that prenatal care allows solving anxieties, bringing peace of mind, as a safe source of information. In this scenario, health professionals stood out as important to meet their demands. Faced with health needs, they aimed at the figure of the professional who provides embracement, guides, and addresses subjects of interest during consultations:

*On the Internet! Now it’s motherhood, everything related to my motherhood, but sometimes I don’t even read that much, because there are studies saying one thing, studies saying another, we are left with even more doubts. I’d rather ask here* [at PNAR]*, because I know it’s more correct and I feel reassured! (G10)*

*As soon as I found out, Google killed me!* [Power of expression to refer to erroneous information, with which she had contact on the internet]*. I got nervous, then thought more calmly, I’m going to Santa Casa to ask. I came here after two days, I got a consultation quickly, and I was well guided, I talked to the doctor here, the doctor there, I tried to find out about other people who had the same problem. This way I understood more, I had the courage to go to Google again, but then I already went to the right sources, as I was taught, and I leave it to God, I know he is ahead of everything! (G27)*

Dialogue and sensitive listening were highlighted as essential functions of professionals, considering that such attitudes play a fundamental role in coping with difficulties, resulting positively in prenatal care and allowing the creation of effective bonds between staff and users, bringing about a feeling of safety and confidence in professional behaviors, with consequent encouragement to follow prescriptions and therapeutic and/or prophylactic recommendations.

## DISCUSSION

### Category 1: Knowledge and Meanings Attributed to High-Risk Pregnancy (Affective Dimension)

To explain the complications generated and multiple effects of HRP, the participants anchored it in negative affects and feelings, highlighting how significant the high-risk diagnosis can be for them, generating negative feelings and sadness.

A study carried out with pregnant women in Paraná also showed that women with HRP are vulnerable to emotional instability and negative feelings, causing a feeling of discomfort and difficulties in accepting this diagnosis, directly affecting their health^(13)^. In a study carried out in a maternity hospital in the United Kingdom^(14)^, most pregnant women expressed fear about childbirth and the baby’s well-being, but the risk was generally accepted as part of the psychological pregnancy strategies they used to deal with their apprehensions, strategies that probably helped them to cope with the risk, projecting better outcomes for themselves and their babies. This finding differs from this study, as feelings of fear, sadness, and concern were highlighted, making them not settle for the pregnancy.

It should be noted that the history, the position of the subject in the group, and the affections corroborate for the (con)formation of the social object and, for this, it must be important for the group representing it^(9)^. Feelings and affections are the basis for building the SRs, supported by memory and experience, because whenever a phenomenon is mentioned, it is talked about from the point of view of individuality, of group belonging, and the feelings generated, evoking practices in relation to it, mutually interweaving knowledge, feelings and affections, reinventing the subject and re-elaborating the object^(15)^.

The psychological aspects in HRP require great investment and psychic processing work. The maternal experience becomes more challenging due to the emotional fragility in which she finds herself at that moment, the increased risk, in addition to other emotions that are connected to her clinical condition^(16)^.

Study^(17)^ carried out with GAR in teaching hospitals in Iran showed that these pregnancies are accompanied by many sensations and profound changes in the mental structure, influenced by perceptions, expectations and previous experiences, requiring adaptation strategies in the context in which the risk occurs.

A study^(18)^ carried out in the metropolitan region of Porto Alegre, highlighted that many mothers used aspects of the personality of the previous children as an essence to think about the characteristics of the fetus and reported these experiences as a basis for care after birth, emphasizing that, even though each pregnancy is unique, the experience linked to the sensations already felt, made them feel calmer to overcome that moment.

However, given the risk diagnosis, pregnancy becomes an unusual and conflicting event. When addressing experiences of this pregnancy, they feel destabilized when they learn that they are giving birth to a child at risk. The impact that the news promotes, the need to (re)organize life and the deprivation of certain resources are associated with insecurity and fear^(19)^.

### Category 2: Fear of the Future and Importance of Family Support (Affective Dimension)

Pregnancy is a physiological process and everything is expected to happen naturally. Thus, HRP escapes the so-called “normal”, it is something unfamiliar, and has to be incorporated into the lives and knowledge of the pregnant women, which characterizes one of the functions of the SR, elaborating HRP and making it understandable in their world, meaning an experience involved in complications, whose death and loss were presented as real and feared possibilities^(8)^.

Therefore, the explanations and fears were anchored in memories of traumatic experiences in previous pregnancies, associated with the death/loss of the baby and the impossibility of sustaining the pregnancy, findings that are in line with meta-synthesis performed with qualitative studies^(20)^, which identified women’s experiences of pregnancy, labor and birth as distressing, contrary to expectations, building meanings anchored in fear and death.

It is worth mentioning that the diagnosis of HRP, as well as the experience of a borderline situation between life and death, brings a feeling of fear and loneliness caused by stress, and the perception of risk is effective in the attitude towards the treatment, maternal decisions in pregnancy and adherence to medical procedures and recommendations^(17)^.

A study with primiparous women in a hospital in the inland area of Rio Grande do Sul^(16)^ pointed out that, due to the fact that their pregnancies are of high risk, they reported that the delivery generated fear and anxiety, because in addition to pain, it would be the moment when they would know if the babies would be born healthy, since they all showed fear of diseases and malformation, results that are close to the findings of this study. The objectification of the healthy, naturally expected baby was set up, whose conception originates in the mother’s mind and can be represented by the imaginary baby, built throughout the gestation, carrying the parents’ desires and projections, attributing desired aspects such as sex, color of eyes, hair type, among others^(18)^.

In this reasoning, it should be noted that the modification of the abstract, idea-concept, to the figurative model, is part of the objectification process and such model has the function of presenting a common point between the different knowledge, translating the real representation of the object and associating the elements in a dynamic that is proper to this object^(9)^. However, starting from the premise that the SRs can undergo modifications, one understands why the materialization of the baby imagined as healthy, in the face of HRP, became one of a baby with health problems, generating anguish.

Faced with these fears and anxieties, family support contributed to the formation of health care concepts and practices for pregnant women, as they were part of their historical and sociocultural context, feeling emotionally supported, encouraged to carry out prenatal consultations and seek proper care. This way, the idea that this support has beneficial effects on the results of pregnancy and childbirth is emphasized, being fundamental for them to experience the new process with peace of mind^(21)^, since significant/affective people can help them face this moment that involves constantly contradictory feelings^(16)^.

Making this explanation in the light of the SRT requires remembering that the SR are inserted in a pre-existing context, full of traditions, values, and beliefs that are anchored to the object that is represented, which can be reincorporated into explanations familiar to the individual and his group, this composition/exchange being essential for the construction of consensual thoughts and sharing with the group, which will allow social belonging^(9)^.

### Category 3: Changes in Health and Daily Habits (Biological Dimension)

SR is understood as knowledge with a practical objective in the construction of social reality^(10)^, involving practices, beliefs, behaviors and taboos, relating to anthropological, cultural, socioeconomic and psychological aspects in each person’s environment. Eating behavior, in its turn, is understood in the attitudes of eating practices together with sociocultural attributes related to food or the act of eating^(22)^, generating a strong impact on everyday life when it requires changes.

Therefore, in addition to physiological changes reflecting on food choices, the pregnant woman is subject to new knowledge or beliefs, either familial, cultural, biomedical or from another source, but which are included in behavior and eating habits during this period^(22)^.

In Brazil, prenatal care includes in its actions eating habits, monitoring of weight gain during pregnancy, and nutritional and/or medication guidelines until breastfeeding, demanding greater attention and strict care in HRP^(1)^. Therefore, the reified knowledge was mobilized by the pregnant women to build their representations, through the guidelines/explanations made by the health professionals and incorporated by the women.

The perception of changes in daily life and care needs, which affected the ability of performing daily activities, requiring rest and closer monitoring, are compliant with the Systematic Review^(19)^ about the emotional and psychological experiences of women in high-risk pregnancies, which showed pregnancy and hospitalization as a new condition, which distanced them from their daily lives and brought them closer to the risk condition, by requiring emotional and structural adaptations, distancing them from their home, work, and family.

Historically, the anchoring of domestic tasks as a female role refers to the stereotype of women and gender ideology that places them as home carers, linked to the family sphere and the domestic environment, where there are commitments and specific tasks of that daily life, such as cleaning the house, cooking and taking care of children and husband^(23)^, which seems to determine the tasks considered traditionally feminine, generating a differential meaning about domestic tasks for men and women. Thus, the impossibility of performing the domestic role put the pregnant women in conflict, starting to objectify the figure of someone incapable, as the novelty of this pregnancy forced profound changes.

Thus, for the operationalization of care practices, from this perspective, it is necessary to establish another look and care contexts governed by expanded health practices, which allow health services to approach the diverse set of issues in which women’s health needs are located. Their quality of life is not only a reflection of choices, feelings, and perceptions, but also the result of policies and social constructs imposed on them^(19)^.

### Category 4: High-Risk Prenatal Care: Challenges and Meanings of High-Risk Pregnancy (Sociocultural Dimension)

Prenatal care is a space of unique construction, influenced by the pregnant woman’s socio-familial set and the performance of health professionals. Therefore, their references and relationships should be considered, as they directly reflect on adherence to prenatal care, understanding of care, and care provided^(24)^.

Other aspects are also indispensable in the search for humanized care in prenatal care, emphasizing the importance of searching for strategies to facilitate access to health services and reduce waiting times^(25)^.

The pregnant woman’s perspective on this assistance can provide adaptations, since the needs perceived by them directly reflect on the way they adhere to preventive and therapeutic actions^(26)^. Common barriers to obtaining timely antenatal care include lack of access to transportation, financial difficulties in getting to health services, and paying for care^(27)^. These barriers, along with cultural beliefs, were also pointed out in this study.

However, despite the barriers and difficulties, it was clear that the SRs about the *PNAR* were built based on positive aspects regarding care, seen as essential for favorable outcomes in childbirth and postpartum. Thus, this prenatal care has to be developed in a harmonious and trusting environment, allowing the expression and verbalization of apprehensions and doubts, guaranteeing safety in the care received^(23)^.

It is in this follow-up that doubts are clarified and actions are taken to prevent injuries, and it is important that professionals recognize the dimension of the care they provide, seeking to work more and more in a humane and embracing way^(27)^.

Because this is a moment of great vulnerability, numerous questions in this period, in the face of concern and insecurity, cannot be answered at the time of diagnosis, requiring time, sometimes endless, generating fears for often not having adequate information and knowledge about their health status^(28)^, with the *PNAR* generating information, arousing curiosities and interest in pregnancy issues.

It is at this moment that digital sources are present in their midst, becoming means of health information and a new phenomenon to be incorporated into everyday life and common sense. Internet research was carried out on a large scale, but one of the challenges for users was the inability to judge the accuracy of the data and images posted. Although they considered some information reliable, much of what was conveyed could be outdated or wrong, leading to incorrect beliefs, driven by inexperience or the desire to share their experiences with others^(29)^.

SRT propositions invite us to reflect on the importance of SRs and their inherent role in mass communication, as social knowledge allows the community to process knowledge conveyed by the media, transforming it into impersonal/public property, which allows each individual to use it consistently with the community’s values and motivations^(9)^.

In this process, they sought, in health consultations, to reaffirm the information obtained in the technological tools^(30)^. In this search for scientific knowledge, they approached reified knowledge, guided by health professionals. The transition of fields of knowledge happens through beliefs, myths, and popular knowledge, which do not present a hierarchy, signal knowledge, purposes, indicate pluralities in the way of thinking and meet the context in which they are produced, even belonging to different fields^(10)^.

In this regard, prenatal care proved to be the right time to develop protective and educational actions, using dialogue, bonding, and listening to pregnant women and their companions, aiming at approximation, strengthening knowledge and clarifying doubts, considering that the results achieved by the multidisciplinary team can offer, in addition to clinical care, emotional and educational support in moments of care^(7)^.

Therefore, this care should not be carried out in a technical way, but through sensitive listening from the perspective of humanization. Nurses shall play a significant role so that pregnant women effectively recognize them in the health team, since by being directly and integrally assisting them, they become fundamental in favoring their well-being, based on dialogue and trust, allowing them to experience this process more calmly and confidently^(23)^.

The limitations of the study are the low possibility of generalizing the representations discussed here, as it was developed with pregnant women from a specific region, where imaginaries and knowledge are strongly influenced by sociocultural factors. Due to the peculiarities and biopsychosocial repercussions of HRP in the daily lives of pregnant women and their families, it is stated that individual interviews, as a data collection technique, did not provide a global understanding of the SR, which is why different studies should be carried out with this public, to know, interpret, and discuss other aspects of the phenomenon. Furthermore, despite the improvement of the data, it is understood that the lexical analysis with IRaMuTeQ may have limited, to some extent, the apprehension of the nuances and subjectivities of the testimonies.

However, the results presented can contribute to a better understanding of the genesis of the representations in this group and support similar studies, since the SRT allows capturing the interpretation of the subjects and the understanding of attitudes and behaviors of social groups, thus being able to lead to health care and nursing that goes beyond the biomedical model.

## CONCLUSION

The pregnant women’s SRs about the HRP revealed negative affects and feelings, an event considered unusual, generating limitations and discomforts, requiring adaptations. Family support was of paramount importance for them and the *PNAR* was a propitious moment for establishing bonds with the health professional, seen as fundamental for their adherence to the care offered.

The SRT was relevant to access this unique way of understanding the pregnant women, because, based on its dimensions, it was observed that HRP is a complex, singular, dynamic and multidimensional event, with SRs being configured from the knowledge and experiences shared in the daily life of this group, full of changes and fears, influenced by common sense knowledge, even conveyed by the media, and reified.

Accessing this knowledge can elicit reflections from health professionals for a humanized and comprehensive care, focused not only on clinical conditions, but also on psychosocial aspects, bringing contributions to HRP assistance, with the nurse having a primordial role as a member of the health team, to identify the demands of pregnant women and provide individualized care.
